# Sociodemographic, income, and environmental characteristics of individuals displaying animal and object hoarding behavior in a major city in South Brazil: A cross-sectional study

**DOI:** 10.14202/vetworld.2021.3111-3118

**Published:** 2021-12-14

**Authors:** Graziela Ribeiro da Cunha, Camila Marinelli Martins, Maysa Pellizzaro, Christina Pettan-Brewer, Alexander Welker Biondo

**Affiliations:** 1 Department of Health Science, School of Veterinary Medicine, Positivo University, Curitiba, Paraná, Brazil; 2 Department of Nursing and Public Health, Ponta Grossa State University, Ponta Grossa, Paraná, Brazil; 3 AAC&T Research Consulting, Curitiba, Paraná, Brazil; 4 Department of Post-Graduate Program in Collective Health, Institute of Collective Health, Federal University of Bahia, Salvador, Bahia, Brazil; 5 Department of Comparative Medicine, School of Medicine, University of Washington, Seattle, Washington, USA; 6 One Health Brasil Association, Brazil; 7 Department of Veterinary Medicine, Federal University of Paraná State, Curitiba, Paraná, Brazil

**Keywords:** epidemiology, hoarding, population characteristics

## Abstract

**Background and Aim:**

Hoarding cases have not been researched in depth in developing countries, such as Brazil. This study aimed to describe the characteristics of people with hoarding behavior in Curitiba, Brazil.

**Materials and Methods:**

A cross-sectional study was conducted based on complaints about hoarding situations received by the City Hall. The data on sociodemographic, income, and environmental characteristics of individuals displaying animal and object hoarding behavior were obtained and analyzed using descriptive statistics and multiple correspondence analyses.

**Results:**

Out of the 113 hoarding cases reported, 69 (61.06%) were fully assessed. Most of the participants (43; 62.32%) were women, and it was observed that most of the animal hoarding cases were women (p=0.02). The average age was 62.47 years old, and most of them (44; 63.76%) had studied up to the middle school level. People associated with object hoarding belonged to the lower income category (p=0.031). In most cases, the homes had an unpleasant odor (45; 65.21%), and this was prevalent in cases involving women (p=0.004) and animals (p=0.001). The risk of fire (24 [34.78%]) and landslip (9 [13.04%]) was more frequent in the case of object hoarding (p=0.018 and 0.021, respectively).

**Conclusion:**

The description of characteristics of individuals with hoarding behavior may assist in understanding the magnitude of this public health problem in Brazil and shed light on the need to develop studies on the health conditions of people and animals that live in these situations.

## Introduction

In the Diagnostic and Statistical Manual of Mental Disorders (DSM-V), hoarding has been defined as a mental disease that is characterized by a reluctance to discard possessions, regardless of their economic value [1]. The accumulation of these possessions may eventually cause serious obstructions in living spaces and could have harmful consequences for the person, their pets, relatives, and the community [1]. The repercussions of this disorder are more extensive in long-term cases. The extreme clutter arising due to hoarding behavior impacts public health as it can lead to unsanitary community conditions and spread of diseases, particularly zoonoses [2,3]. These unsanitary conditions have an impact on the health of the individual with hoarding disorder and may lead to risks such as falling objects, fire hazards, and fire exit obstructions, which compromise their safety and welfare and lead to social vulnerability [4-9]. Other negative effects are primarily associated with poor physical health, increased risk of injury, exacerbation of chronic diseases, occupational impairment, and social concerns, such as homelessness, social isolation, and economic burden [4,7,10,11]. Family-related frustrations arising due to this behavior may cause conflict within relationships or may even lead to ending relationships. However, these conflicts often lead to the exacerbation of the disorder [3,9]. The conditions in hoarding households may worsen with animal hoarding [2,6] and the added animal noise and odor from feces can lead to an increase in neighborhood complaints [6,8,12,13].

Studies on the object and/or animal hoarding have been conducted in the United States [8,14], Spain [13,15], Italy [16], England [17], and Australia [12,18,19]. These studies have described the characteristics of individuals with hoarding disorder in developed countries based on a variety of epidemiological methods. It is imperative to identify the characteristics of object and animal hoarding cases in Brazil as they may differ due to the economic, cultural, and geographical differences between underdeveloped and developed countries. A comparison between the results of the studies can provide information regarding the similarities and variations observed in developed and underdeveloped countries and this information can assist in determining the specific intervention required.

This study aimed to establish the sociodemographic characteristics and risk factors associated with hoarding behavior in Curitiba, Paraná State, Brazil. It also compares object and animal hoarding cases.

## Materials and Methods

### Ethical approval

The study was approved by the Ethics Research Committee of Health Sciences, Federal University of Paraná (protocol number 1,105,785/15). The Curitiba Secretaries of Health, Environment and Social Assistance gave the consent and permission to access the data collected during official inspections and to conduct the study.

### Study area

This cross-sectional study was conducted from September 2013 to April 2015. The study was conducted in Curitiba (25°25’47” S, 49°16’19” W), which is the biggest city and the capital of Paraná State and has an area of 435.036 km^2^. Curitiba is the eighth most populated city in Brazil, with an estimated population of 1,948,626 in 2020 [20].

### Procedures

The study focused solely on the epidemiological characteristics of hoarding behavior cases. The data were obtained by tracking hoarding complaints received by the municipality central phone line and official inspectors investigated these cases. The residents of Curitiba can use the municipal central phone line to file complaints regarding any problem in the city, including issues related to animals, public health, environment, and social vulnerability. All complaints received were verified in-person by official inspectors and clarifications regarding the complaint were provided to the complainant. A multidisciplinary team of professionals from the Curitiba City Hall investigated the complaints about hoarding behavior. This team was titled as the “hoarding behavior workgroup” and included physicians, nurses, psychologists, veterinarians, biologists, and social services personnel. Each complaint was investigated to determine if it was a genuine hoarding case. Researchers from the Federal University of Paraná trained the workgroup members regarding identification of hoarding cases, and they assisted in approaching individuals and identifying hoarding behavior, as well.

The cases included in this study were based on the observation of the physical consequences of the probable hoarding behavior when the suspected living space was inspected. Furthermore, specific attention was given to conditions that may have affected family members and the community.

The inclusion criteria for probable object hoarding cases were based on the observation of any of the following characteristics: A large number of items that had been accumulated without an apparent purpose, the obstruction of living spaces in the household, and a reported reluctance to dispose of objects. The inclusion criteria for probable animal hoarding cases were based on the observation of any of the following characteristics: Animal accumulation, a lack of health standards, space, nutrition, or veterinary care, and a refusal to surrender the animals. After inspection, a decision was taken if the case should be classified as a probable hoarding behavior case. It is important to emphasize that the inclusion criteria considered the definition of hoarding disorder presented in the DSM-V [1]. However, a clinical diagnosis was not a part of the inclusion criteria.

### Data collection

The characteristics of the participants were collated through a standardized questionnaire used by the official inspectors in their work routine. Specifically, objective questions were used to collect information about the individuals and observational questions were used to collect information about the environment and household conditions. The data collection instrument was adapted from the HOMES^®^ Multi-disciplinary Hoarding Risk Assessment (Oxford University Press, Oxford) [21]. The variables investigated included, gender (man or woman); age, which was categorized as <60 and ≥60 years old; education level, which was grouped into three categories as, till middle school, high school, and college, according to the highest level achieved; monthly income based on the Brazilian minimum wage (MW) (US$ 225.00 or R$ 880.00), with income at the time of study being divided into three categories, that are, ≤1 MW, >1 and ≤3 MW, and >3 MW; and the number of people living in the household, which was divided into two categories, that are, the hoarder lives alone or with one person and the hoarder lives with two or more people. The factors also included information of the presence or absence of self-reporting of physical health problems, such as, high blood pressure and diabetes, and other problems related to assistance from relatives, perception of self-care, and any evident mental impairment other than the hoarding disorder.

The characteristics of the hoarded animals, such as the total number, species (dogs, cats, and others), and general health conditions, were ascertained through visual inspection. The characteristics were classified collectively as “bad,” if most of the animals had clinical signs of any disease, behavioral problems, or low body scores. Animals were classified as “good,” if they did not have any such issues or they were classified as “regular” if their condition was neither “good” nor “bad.” This classification also took into consideration their habitat, that is, if their living space was hygienic or if the area was extremely dirty and had feces and urine. It was cross-checked if the area was routinely cleaned, the handling capacity of the area was ascertained, and it was determined if the animals had free roaming space in the yard, lived inside the house, in individual kennels, in collective kennels, were chained outdoors, or caged.

Household conditions were assessed by the official inspectors during the home visit. The factors considered included their observation of any unpleasant odor, vector proliferation, the evidence of pest infestation (such as standing water, garbage accumulation or pest infestation itself, that is, rats, cockroaches, and mosquitoes), the possibility of fire risk (e.g., no electricity was available, usage of candles, or the house electrical system was compromised), or the risk of landslip (e.g., when the accumulation of objects was so extreme that they could fall on the individual or the animals).

### Statistical analysis

Data were initially grouped into object accumulation (i.e., cases in which objects were involved) and animal accumulation (i.e., cases in which animals were involved). Dichotomous variables were used to classify the data (e.g., yes/no and presence/absence).

The R software version 3.1.0 (R Foundation for Statistical Computing, Vienna, Austria) environment was used to conduct statistical analyses [22]. All variables were evaluated using descriptive and univariate analyses with frequencies (simple and cross-tables), 95% confidence intervals (CI) for frequencies, odds ratio estimates and their corresponding 95% Cis, and Chi-square test (significance level=0.05).

To explore multiple associations between the variables, multiple correspondence analysis (MCA) was performed. MCA is an alternative for multiple analyses of qualitative data wherein the intent is to verify associations without necessarily obtaining coefficients, such as in regression models. The main variable was the accumulation of animals and objects and the association with the following variables: Gender, categorized age, categorized income, categorized education, presence of health problems, family assistance, odor, risk of fire, risk of landslip, or risk of vector proliferation. The generated graph was visually interpreted by comparing the proximity and length according to the axes of each variable’s category using the “ca” [23], “FactoMineR” [24], and “factoextra” [25] packages of R [22].

## Results

A total of 226 hoarding complaints were investigated. Of these, 113 (50.0%) were shortlisted as probable hoarding cases as they met the inclusion criteria, 61 (27.0%) were excluded because they did not meet the inclusion criteria, 32 (14.2%) were not found during the inspection visits, and the wrong address had been given by the complainant for 20 (8.8%). The inspectors were able to administer the complete questionnaire for 69 (61.06%) of the confirmed cases. The remaining cases (44; 38.93%) were excluded due to reasons including death, hospitalization, moving, or refusal to participate in the workgroup visit and inspection.

From the ones that were inspected, 25 (36.23%) were classified as only animal hoarding, 30 (43.47%) were classified as only object hoarding, and 14 (20.28%) involved both, animal and object hoarding. Therefore, animals were involved in 39 (56.52%) and objects were involved in 44 (63.76%) hoarding behavior cases.

Forty-three of the individuals with hoarding behavior (62.31%) were women and 26 (37.68%) were men. It was found that a significantly larger number of women (p=0.02) were involved in animal hoarding cases (n=39). The age ranges from 33 to 84 (mean=62.47±11.30). Specifically, 29 (42.02%) individuals were <60 years while the remaining (57.97%) were older. In terms of education level, most of the individuals with hoarding behavior had studied up to middle school (44; 63.77%) followed by those who studied up to high school (16; 23.18%) and college (8; 11.59%). Most individuals with hoarding behavior reported that they lived alone or with one other person (48; 68.56%) and 20 (28.98%) stated that they lived with two or more people at the time of inspection. In terms of income, the largest number of individuals with hoarding behavior reported up to 1 MW (35/69; 50.72%), and this was primarily observed in the case of object hoarding (p=0.031). Detailed information regarding the demographic characteristics is presented in Table-1.

**Table 1:** Sociodemographic characteristics of hoarding behavior cases in Curitiba, Brazil, from 2013 to 2015.

Characteristics	Animals (39 cases)	Objects (44 cases)	Total (69 cases)	
Age (years)				
Mean±SD	62.25±11.76	62.52±10.61	62.47±11.30	
Gender	n (%)	n (%)	n (%)	CI 95%
Female	29 (74.35)	24 (54.54)	43 (62.32)	50.88-73.75
Male	10 (25.64)	20 (45.45)	26 (37.68)	26.25-49.12
Age (age groups)				
0-49	5 (12.82)	6 (13.63)	10 (14.49)	6.19-22.80
50-59	12 (30.76)	12 (27.27)	19 (27.53)	17.00-38.08
60-69	10 (25.64)	14 (31.81)	20 (28.98)	18.28-39.69
70 or more	12 (30.76)	12 (27.27)	20 (28.98)	18.28-39.69
Level of education				
Illiterate	5 (12.82)	5 (11.36)	8 (11.59)	4.04-19.15
Elementary school*	10 (25.64)	17 (38.63)	22 (31.88	20.89-42.88
Middle school*	9 (23.07)	10 (22.72)	14 (20.28)	10.80-29.78
High school*	10 (25.64)	7 (15.90)	16 (23.18)	13.23-33.15
College*	5 (12.82)	4 (9.09)	8 (11.59)	4.04-19.15
No answer	0 (0.00)	1 (2.27)	1 (1.44)	
Monthly income				
≤1 minimum wage	18 (46.15)	27 (61.36)	35 (50.72)	38.93-62.52
>1-≤3 minimum wage	15 (38.46)	10 (22.72)	23 (33.33)	22.21-44.46
>3 minimum wage	6 (15.38)	5 (11.36)	9 (13.04)	5.10-20.99
No answer	0 (0.00)	2 (4.54)	2 (2.89)	
Number of people living in the household				
0	1 (2.56)	1 (2.27)	1 (1.44)	0.00-4.27
The individual with hoarding behavior (IHB)	16 (41.02)	16 (36.36)	27 (39.13)	27.61-50.65
The IHB and one	11 (28.20)	15 (34.09)	21 (30.43)	19.58-41.29
The IHB and 2	8 (20.51)	3 (6.81)	10 (14.49)	6.19-22.80
The IHB and 3 or more	3 (7.69)	9 (20.45)	10 (14.49)	6.19-22.80

* Level of education can be complete or incomplete

In terms of health, 53 (76.81%) individuals with hoarding behavior reported that they suffered from chronic diseases, such as diabetes, high blood pressure, depression, and cancer. Regarding self-care, it was determined that 38 (55.07%) of them lacked personal hygiene and 37 (53.62%) did not show any noticeable mental impairment concurrent with hoarding disorder. In addition, 58 (84.05%) of the individuals with hoarding behavior reported that they received some assistance from their relatives.

The biggest risk factor observed with probable hoarding cases was vector and pest proliferation (61/69; 88.40%), followed by an unpleasant perceptible odor (45/69; 65.21%), which was higher in the case of female hoarders (p=0.004) and animal hoarding (p=0.001). Fire and landslip risks were reported in 24 (34.78%) and 9 (13.04%) cases, respectively. Both fire (p=0.018) and landslip (p=0.021) risks were significantly higher in object hoarding cases (Table-2).

**Table 2:** Environmental factors associated with hoarding cases per type of accumulation in Curitiba, Brazil, from 2013 to 2015.

	Objects		OR (95% CI)	Animals		OR (IC 95%)
	
	n (%)	p-value		n (%)	p-value	
Unpleasant odor						
Yes (n=45)	23 (51.1)	0.003	0.14 (0.04-0.57)	32 (71.1)	0.001	5.98 (2.01-17.79)
No (n=24)	21 (87.5)			7 (29.2)		
Risk of vectors proliferation						
Yes (n=61)	38 (62.3)	0.482	0.55 (0.10-2.96)	36 (59.0)	0.449	2.40 (0.52-10.97)
No (n=7)	5 (71.4)			3 (42.9)	
Risk of fire						
Yes (n=24)	20 (83,3)	0.014	4.37 (1.29-14.86)	10 (41.7)	0.08	0.39 (0.14-1.08)
No (n=45)	24 (53,3)			29 (64.4)		
Risk of landslip						
Yes (n=9)	9 (100.0)	0.015	1.71 (1.38-2.12)	3 (33.3)	0.156	0.33 (0.07-1.50)
No (n=59)	34 (57.6)			36 (61.0)		

**Figure-1:**
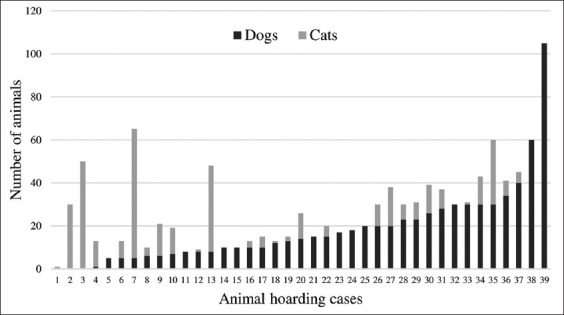
Distribution of dogs and cats per animal hoarding case in Curitiba, Brazil, from 2013 to 2015.

Thirty-nine hoarding cases pertained to animal hoarding and a total of 1104 pets were found. Of these, 722 were dogs (ranging from 1 to 105, mean: 20.05 dogs/case) and 382 were cats (ranging from 1 to 60, mean: 13.64 cats/case). In 11 (28.20%) cases, only dogs were found, and in 3 (7.69%) cases, only cats were found. In 25 (64.10%) cases, both dogs and cats were reported (Figure-1). In 10 (25.64%) cases, the presence of other species, primarily birds, was also reported in the household.

The collective conditions of the animals were considered regular in 17 (43.58%) cases, good in 12 (30.76%), and bad in eight (20.51%) cases. An answer regarding the condition was not obtained in 2 (5.12%) cases. Regarding the environment, animals were found to be living freely in the yard in 36 (92.30%) cases, inside the home in 21 (53.84%) cases, in individual kennels in 10 (25.64%) cases, in collective kennels, in 10 (25.64%) cases, chained outdoors in 10 (25.64%) cases, and caged in 5 (12.82%) cases.

**Figure-2:**
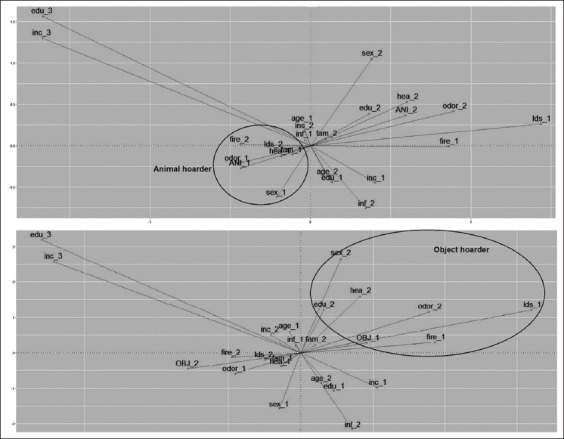
Multiple correspondence analysis between animal and object hoarding cases in Curitiba, Brazil, from 2013 to 2015.

MCA with selected variables indicated an association of animal hoarding behavior cases with women, people with health problems, those who had family assistance, presence of unpleasant odor, and no risk of landslip (Figure-2). The same analysis showed an association of object hoarding behavior cases with men, without related health problems, high school level of education (complete or incomplete), report of local risk of fire or landslip, and no report of unpleasant odor (Figure-2).

(ANI_1) Animal hoarding; (ANI_2) No animal hoarding; (OBJ_1) Object hoarding; (OBJ_2) No object hoarding; (sex_1) Woman; (sex_2) Men; (inc_1) Income less or equal than 1 MW; (inc_2) Income between 1 and 3 MW; (inc_3) Income more than 3 MW; (edu_1) Until middle school; (edu_2) High school; (edu_3) College; (hea_1) With health problem; (hea_2) No health problem; (fam_1) Family assistance; (fam_2) No family assistance; (odor_1) Unpleasant odor; (odor_2) No unpleasant odor; (fire_1) Fire risk; (fire_2) No fire risk; (lds_1) Landslip risk; (lds_2) No landslip risk; (inf_1) Risk of vectors proliferation; (inf_2) No risk of vectors proliferation.

## Discussion

This study assesses object and/or animal hoarding characteristics observed in individuals in a major city in Brazil. The results highlight some important information regarding implications of these cases. Considering the complexity of this public health problem, the data could assist in developing a multidisciplinary intervention approach that would be helpful in managing the environmental, animal, and human health issues that arise due to hoarding.

The observation that instances of hoarding behavior in women are more prevalent (54.54%) (Table-1) also has been reported in the previous study conducted in Italy (68.9%) [16] and in two studies conducted in Boston, USA (71.4% [26] and 79.8% [27]). The same was observed in animal hoarding cases (Table-1) and is also reflected in other studies conducted in New South Wales, Australia [12] (72.4%), and in two studies conducted in the USA (76% [8] and 83.1% [28]). The findings of the previous studies could be attributed to the fact that the samples included in investigations are primarily those of females [29] or may represent gender differences in seeking help for the hoarding problem [7]. This study also found that the frequency of hoarding behavior in females was statistically significant. However, due to the sample recruitment method used, it cannot be assumed that hoarding behavior is more prevalent in women than men.

The average age of the people in this study was 62.47 years old (Table-1), which was higher than the previously reported ages found in Italy (36.2 years) [16], London (48.8 years) [17], three different studies in Boston (49.18, 53.3, and 54.8 years) [11,26,27], and in New South Wales (54.8 years) [12]. The age identified in this study is similar to the average age found in the reports from Spain [13]. As individuals who are 60 years or older are considered elderly in Brazil, the percentage (57.97%) of people in this age group was higher in this study than in the previous studies pertaining to animal hoarding. Another study conducted in Brazil also found the predominance of elderly people in animal hoarding cases [30]. This result could be attributed to the lack of family or public assistance when symptoms of hoarding behavior begin or a lack of early identification of possible cases, as the symptoms may have begun in childhood and adolescence and become more severe as age increases [29].

The education level most reported herein was up to middle school (63.76%) (Table-1), which is equivalent to 8 years of education in Brazil. Similar results were found in another study in Brazil [30]. Studies in other countries found that the average education was 12 years (high school) in New York [31], 17.8 years in Boston [26], 17-18 years in Italy [16], and individuals with a university diploma in London [17]. These conflicting data may be attributed to the fact that there are a large number of school dropouts in Brazil and other developing countries. This could be because of the lack of investment in education by the government. This information may have a significant impact on hoarding characteristics worldwide and should be considered when applying a specific care protocol in each locality.

Half of the individuals with hoarding behavior (50.72%) reported that they had an income of until one Brazilian MW per month (US$ 225.00), and only a few people (13.04%) reported that they received more than US$ 675.00 each month (Table-1). The results of this study indicate a lower financial capacity than what was previously reported in New York (US$ 540.00 up to US$ 3599.00/month) [31] and in Baltimore (1666.00/month) [32]. However, 78.57% of hoarding cases in Spain were described as having a borderline financial situation [13]. Furthermore, the income of animal hoarders tends to be more concerning as it is also used to purchase animal feed. A previous study demonstrated a high prevalence of low income (1-2 MW) among animal hoarding cases in Brazil [30]. This finding leads to economic concerns for the diseased, marginalized, and unassisted population [11,17].

The frequency of individuals with hoarding behavior that reported to be living alone (41.02%), in this study, was lower than that in the USA (55.6%) [8] and Spain (83%) [13]. The percentage of animal hoarding cases who lived with another person was higher (28.20%) in this study than in the USA (14.8%) [8] (Table-1). An Australian study found that 64% of female and 37.5% of male hoarders lived with someone else [18]. However, most people studied herein reported that they received assistance from relatives, living alone or with just one person may place the individual in a vulnerable situation, such as lack of availability of food, health care, and good environmental conditions.

A high percentage of individuals with hoarding behavior reported health problems (76.81%), which were mainly chronic diseases. This was similar to the 63.6% of hoarders who met the diagnostic criteria for chronic medical health conditions in Boston [11] but differed from the 63.2% who reported good health in London [17]. As these subjects were elderly people with chronic diseases and had physical health problems, it can be assumed that they received some health assistance, primarily from the decentralized public health system in Brazil. Thus, it may be inferred that hoarding disorder was either unnoticed or neglected by public health services. It is imperative to reinforce that early mental health assistance could improve the chances of successfully treating people with hoarding behavior.

Although the household conditions evaluated herein were based on subjective observation of risks, they suggest an approximation of the real risk found in the cases and should be considered with a multidisciplinary intervention approach. Although the household conditions evaluated herein were based on a subjective inspector’s observation of risks, they suggest an approximation of the real risk found in the cases and should be considered in a multidisciplinary intervention approach.

The risk of vector proliferation was documented in 88.40% of the cases. This could be attributed to unsanitary environments, mostly in animal hoarding cases. This observation is similar to that of another study wherein it was reported that in 88% of the complaints, unhealthy living conditions were found [6]. For this reason, hoarding cases are considered a public health hazard that compromises the entire surrounding community [5,7,9]. This condition may increase the risk of health problems and zoonotic diseases, such as leptospirosis, gastrointestinal infections, and venomous animal accidents, among others. Studies establishing the health risk to which individuals with hoarding behavior and their surrounding community are exposed should be conducted to provide appropriate subsidies for early intervention in hoarding cases.

An unpleasant odor was reported in 65.21% of the cases. An odor indicates unsanitary environmental conditions. This result is similar to previously described results, in which odor was reported in 53% of hoarding complaints in Massachusetts [6]. Moreover, the worst environmental conditions were likely to be observed among female hoarders and those in which animals were hoarded. Such findings are in accordance with a study performed in Massachusetts, which established that the living conditions of animal hoarders are less sanitary than object hoarders [6].

In 34.78% of the cases analyzed in this study, a risk of fire was observed. This percentage was lower than what has been previously reported in hoarding cases in Massachusetts (67%) [6] and across the USA (70.4%) [28]. The landslip risk reported (13.04%), mostly in object hoarding behavior cases, was also lower than the previously reported percentage of 80.2% in animal hoarding cases across the USA [28]. Both fire and landslip risks were more prevalent among object hoarding behavior cases, indicating that clutter may become fuel, obstruct fire exits, absorb the water used to combat a fire, and cause fatal injuries due to falling objects, all of which could lead to disastrous consequences [4].

It was observed that a significantly higher number of women (p=0.02) were involved in animal hoarding cases, and this finding is similar to the results of another study conducted in Brazil [30]. This could be possibly attributed to the greater attachment between women and companion animals and the greater involvement of women in social causes associated with animal rights and welfare [33]. Although animal hoarding cases involved the hoarding of dogs and cats together (64.10%), cases in which there were more dogs involved fewer cats and vice versa (Figure-1). This is not the same as the findings of a study conducted in Spain, in which predominantly dogs were hoarded (58.33%) [13], and another research in Australia in which only cats were hoarded (63%) [19]. The general condition of animals involved was classified as regular in 43.58% and good in 30.76% of cases. Contrastingly, 83.33% of cases in Spain presented animals in poor condition [13], and in 90% of cases in Australia, the animals were found in unsanitary conditions [19]. It cannot be assumed that individuals with hoarding behavior in this study provide better animal care and the outcome could, therefore, be attributed to differences in the classification method. The high rate of animals observed living inside the home (53.84%) indicates closer contact with people, and this may increase the likelihood of and facilitate the transmission and spread of zoonotic diseases and occurrence of injury [2]. The human-animal bond observed in such hoarding cases demonstrates the importance of having a veterinarian in this multidisciplinary workgroup to help manage cases.

The MCA applied in the present study reinforced the significant association between animal hoarding cases and the female gender, report of health problems, family assistance, residence with an unpleasant odor, and lack of landslip risk (Figure-2). The analysis also indicated that education, income, vector proliferation, and risk of fire were not associated with animal hoarding cases. This implies that these factors may not be relevant while evaluating animal hoarding behavior when applying MCA to similar populations.

In the MCA, object hoarding cases showed a significant association with men, as well as, with a lack of reported health problems, high school education, risk of fire and landslip, and no report of unpleasant odor (Figure-2). This analysis indicates that income, age, and risk of vector proliferation were not associated with object hoarding cases and that these factors may not be relevant in the evaluation of object hoarding behavior under the same conditions.

## Conclusion

Animal hoarding cases were primarily associated with the female gender, and the main associated risks were vector proliferation and unpleasant odor, which indicated unsanitary conditions in the household environment. Elderly women, those with a low income and level of education, individuals living alone or with another person, those with health problems, and individuals receiving some assistance from relatives were more likely to hoard animals. Men were mostly associated with object hoarding cases and these involved the risk of fire and landslip. Adult men, individuals not suffering from health problems, and those with a low level of formal education or low income were more likely to hoard objects in Curitiba. The findings of this study have established the main characteristics of individuals displaying animal or object hoarding behavior in Curitiba. The data may assist in the development and establishment of a specific multidisciplinary care protocol for these individuals and their animals, primarily because economic and cultural diversity influences case intervention.

## Authors’ Contributions

GRC, MP, CMM, and AWB: Conceptualization and design of the study. AWB: Project administration and supervision. GRC and MP: Investigation and data collection. GRC: Data interpretation and original draft preparation. CMM: Statistical analyses and data interpretation. GRC, MP, CMM, CP, and AWB: Drafting of the manuscript and critical review. All authors read and approved the final manuscript.
